# Floral ultraviolet absorbance area responds plastically to ultraviolet irradiance in *Brassica rapa*

**DOI:** 10.1002/pei3.10091

**Published:** 2022-09-29

**Authors:** Liberty A. Gray, Sandra Varga, Carl D. Soulsbury

**Affiliations:** ^1^ School of Life and Environmental Sciences University of Lincoln Lincoln UK; ^2^ Institute of Evolutionary Biology, School of Biological Sciences University of Edinburgh Edinburgh UK

**Keywords:** *Brassica rapa*, plasticity, ultraviolet radiation, UV bullseye, UV exposure, UV‐B

## Abstract

Solar ultraviolet (UV) radiation is known to have significant effects on the development and performance of plants, including flowers. In multiple species, UV‐absorbing floral patterns are associated with environmental conditions such as the solar UV exposure they typically receive. However, it is not known whether plants can increase the UV‐absorbing areas found on petals plastically when in a high‐UV environment. We grew *Brassica rapa* at three different UV radiation intensities (control, low, and high) and under two exposure duration regimes. We removed petals from flowers periodically during the flowering period and measured the proportion of the petal that absorbed UV. UV‐absorbing areas increased when plants were exposed to longer periods of UV radiation, and at high UV radiation intensities. UV‐absorbing area of petals of the UV intensity treatments decreased over time in long exposure plants. This study demonstrates that flowers can potentially acclimate to different UV radiation intensities and duration of exposure through an increase in UV‐absorbing areas even after a relatively short exposure time to UV. Such a rapid plastic response may be especially beneficial for dynamically changing UV conditions and in response to climate change.

## Graphical abstract

Flowers can potentially acclimate to different ultraviolet (UV) radiation intensities and duration of exposure through an increase in UV‐absorbing areas even after a relatively short exposure time.

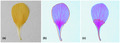

## 1 INTRODUCTION

For many years, we have known that ultraviolet (UV) stress is induced by exposure to relatively high intensity of UV radiation and/or low intensity of photosynthetically active radiation (PAR; Ballaré et al., 2011; Jansen et al., 1998). This can translate into a reduction in biomass accumulation, reduction in photosynthesis, damaging large macromolecules such as DNA and other proteins, and inducing significant oxidative stress within the vegetative tissues of plants (Frohnmeyer, 2003; Jansen et al., 1998; Rozema et al., 1997).

UV‐B is a portion of solar radiation that is biologically active, and can induce profound changes in gene expression, physiology, and morphology of plants (Jansen, 2002). Most of the solar UV‐B radiation that reaches the Earth's surface is absorbed by the stratospheric ozone layer, and atmospheric ambient UV‐B radiation varies depending on factors such as season, latitude, altitude, and time of day (McKenzie et al., 2007). More importantly, it is becoming well established that ozone depletion coupled with climate change is increasingly modifying the timing and duration of UV‐B radiation exposure for plants (Bornman et al., 2019). Recent research indicates concurrent exposure to UV‐A and PAR can minimize the detrimental effects of high UV‐B (Verdaguer et al., 2017). It is now becoming clear that broad spectrum UV radiation may influence plant metabolism and other important processes (Jansen & Bornman, 2012; Jenkins, 2009), and accumulated evidence suggests that, under realistic exposures scenarios, the negative effects of UV‐B on plant performance are indeed relatively minor or non‐existent (Fiscus & Booker, 1995).

In most instances, low UV intensity can be characterized as regulatory or “eustress” to plants (i.e., good stress, Hideg et al., 2013), inducing plastic responses in both vegetative and reproductive plant tissues (Jansen, 2002; Llorens et al., 2015). In some cases, plastic responses to elevated UV such as rapid increase in flower production and earlier anthesis (Torabinejad et al., 1998; Ziska et al., 1992) are suggestive of a strategic stress response, increasing reproductive output before yielding to the stressor for example (Wada & Takeno, 2010). A good example of these plastic responses includes increased accumulation of UV‐absorbing compounds (such as flavonoids) in the pollen walls of plants grown in UV‐B treatments that are significantly higher than typical ambient levels (Demchik & Day, 1996), or increased accumulation of anthocyanins and flavonoids in flowers (Ben‐Tal & King, 1997; Hennayake et al., 2006), overall producing petals with more intense color than petals of flowers grown in UV‐absent conditions (Ben‐Tal & King, 1997).

Research into UV floral pigmentation has identified UV‐B as a potential selective agent of the UV‐absorbing areas (bullseye) floral pattern (Koski & Ashman, 2015). The bullseye is a pattern formed by the arrangement of UV reflecting petal apices and UV‐absorbing petal bases (Guldberg & Atsatt, 1975; Harborne & Nash, 1984). UV bullseyes, and their ecological development, have been traditionally understood as the result of pollinator‐driven interactions. Much of the floral diversity we see today, and in particular in UV bullseye pattern, indicates that UV bullseyes function to aid pollinator perception, orientation, visitation, foraging efficiency, and ultimately plant fitness (Koski & Ashman, 2013; Rae & Vamosi, 2013; Sheehan et al., 2016). However, the recent findings that *Argentina anserina* (Rosaceae), a globally widespread plant, produce flowers with a higher proportion of UV‐absorbing area (UV_proportion_) with decreasing latitude (UV‐B irradiance is greater at low latitudes), and that the UV_proportion_ of 177 species within the Potentillae correlated with broad geographic variation in UV‐B irradiance (Koski & Ashman, 2016) suggests that UV‐B could be an important overlooked abiotic driver of phenotypic variation in plants (Koski & Ashman, 2015), even though this has not been started to be experimentally tested until recently (Finnell & Koski, 2021).

Flowers producing UV bullseyes with greater UV_proportion_ are thought to be the object of selection because they significantly improve their male fitness in elevated UV‐B environments (Koski & Ashman, 2015). Flowers with more UV reflectance are hypothesized to experience decreased pollen viability via diffuse reflection of UV onto pollen‐bearing anthers; conversely, flowers with larger bullseye protect pollen by absorbing UV radiation (Koski & Ashman, 2015). The extent of protection is such that the germination rate of pollen from flowers in relatively high UV conditions, but with large bullseyes, is comparable with that of flowers grown in UV‐absent conditions (Koski & Ashman, 2015; Peach et al., 2020), even though this may not be ubiquitous in plants. It should be also noted that these previous studies could not elucidate whether UV‐B irradiance was the ultimate factor mediating this response in the wild. Many other environmental variables also change with latitude (i.e., temperature, PAR, seasonality) and there are strong links between UV‐B radiation and climate change (Bornman et al., 2019). Therefore, in the present study, we tested whether UV_proportion_ variation directly reflects changing UV‐B radiation intensity and duration of exposure during plant growth using *Brassica rapa* flowers, a species known to show UV_proportion_ variation among genotypes and cultivars (Yoshioka et al., 2005). Because previous studies have indicated that UV bullseyes are the target of selection, functioning in part to protect pollen from harmful levels of UV‐B damage (Koski & Ashman, 2015; Peach et al., 2020), it now seems pertinent that we examine whether plants have the capacity to increase the UV‐absorbing areas of their petals in response to increased exposure and intensity of UV‐B radiation. This capacity may be especially important if the pace of climate change exceeds the speed that wild plants can evolve these features. We predict that UV‐absorbing areas are phenotypically plastic, and expect that UV_proportion_ will be greater in the petals of plants exposed to high UV‐B radiation intensities. *B. rapa* is insect‐pollinated and represents a valuable agro‐economic resource and improved knowledge of its floral response(s) to UV‐B may inform about the appropriate use of genotypes and cultivars on a regional to global scale, where UV radiation varies according to both latitude and season (Herman, 2010).

## 2 MATERIALS AND METHODS

### 2.1 Study species

Field Mustard *B. rapa* L. (Brassicaceae) is an annual crucifer native to Europe. It has been extensively cultivated, and is now distributed globally, and found on waste ground, roadside, and both river and stream bankside habitats. To the human eye, the flowers have four bright yellow petals which are arranged symmetrically, with six stamens in the center. In addition, *B. rapa* has a clear UV‐bullseye (Yoshioka et al., 2005). To test whether UV is a driver of phenotypic plasticity in a specific floral trait (UV bullseye), we used the rapid‐cycling *B. rapa*, developed by Williams and Hill (1986) via several cycles of selection. Compared to their wild relatives, rapid‐cycling *B. rapa* plants flower earlier (~18 days from planting), producing up to 20–25 flowers per plant.

### 2.2 Experimental design: plants

**FIGURE 1:**
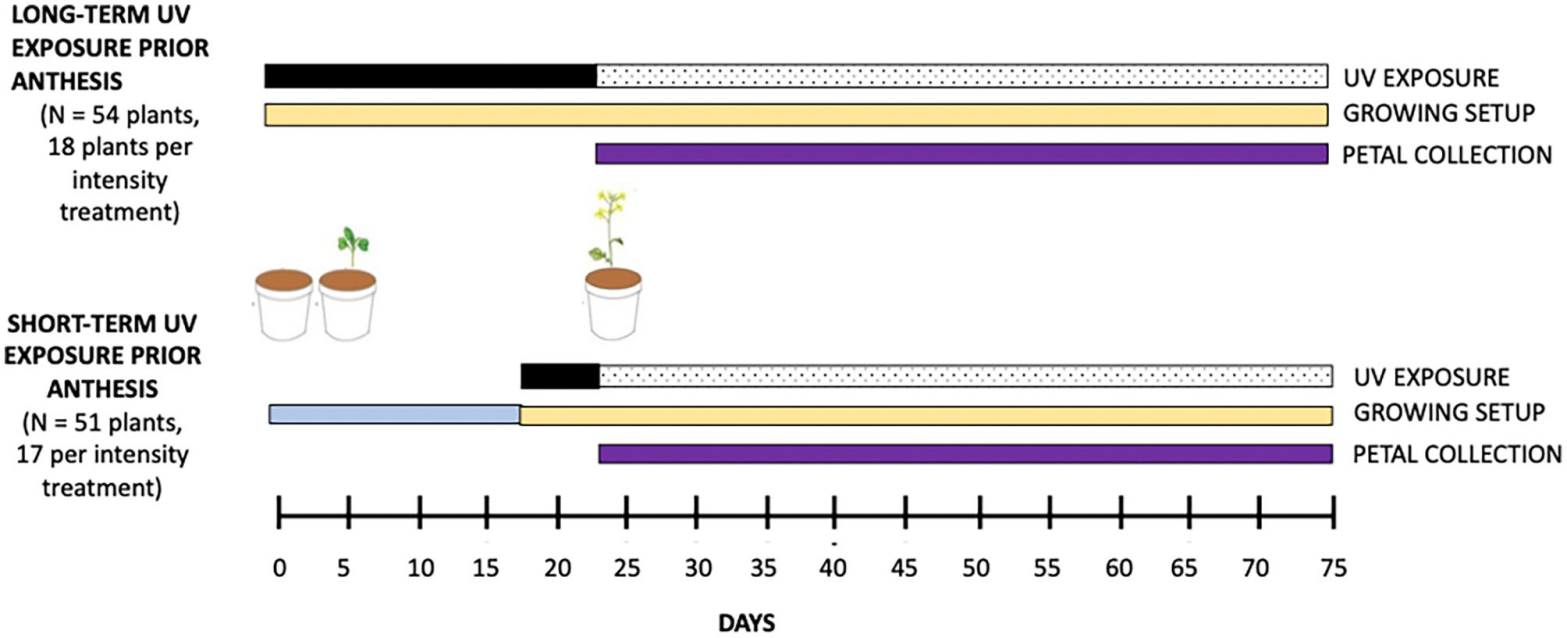
Schematic diagram showing the duration (black boxes) of the long‐ (24 days) and short‐term (6 days) UV exposure prior anthesis in the experimental plants. Long‐term UV‐exposed plants were grown exclusively in the growth chambers (yellow box) for the duration of the study. Short‐term exposure plants were grown for 18 days in a glass greenhouse (blue box) before moving them in the growth chambers (yellow box). UV, ultraviolet.

Individual *B. rapa* plants were exposed to one of two periods of UV exposure prior to anthesis: (i) long‐term UV exposure (~24 days) and (ii) short‐term (~6 days) UV exposure (Figure 1).

**FIGURE 2:**
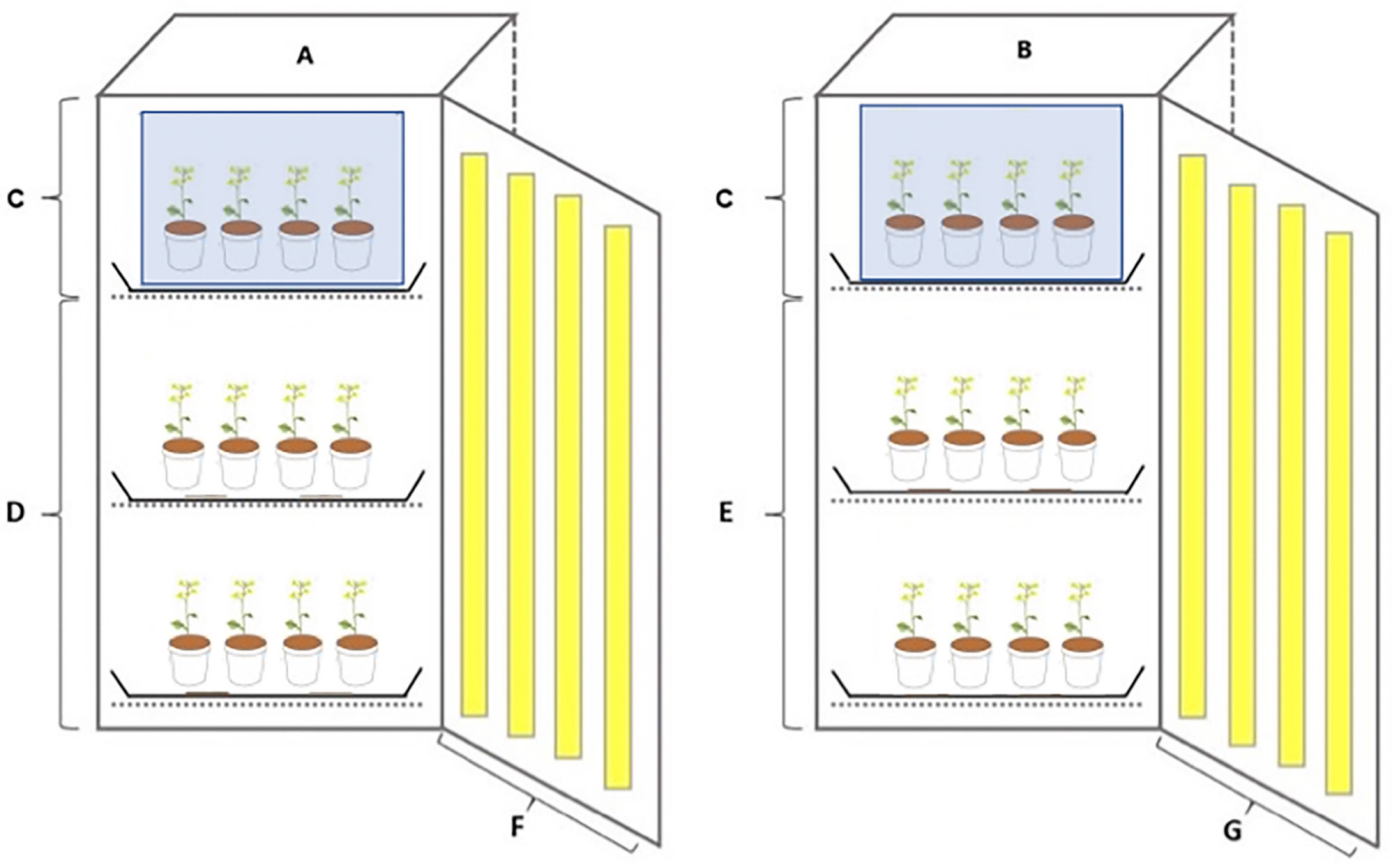
Schematic diagram of the plant chambers used to simulate UV‐B intensity treatments for both long and short exposure experiments. (A and B) represent low and high UV‐B intensity chambers, respectively. In each chamber, control plants were placed within identical custom‐built Perspex® VE grade UV‐absorbing acrylic boxes that restricted UV light transmission from 250 to 400 nm (C). (D and E) represent low and high UV‐B intensity treatment plants, respectively. In the low UV‐B intensity treatment, 15 × 36 W 6% UV‐B bulbs were fitted into the front and side panels of the chamber (F); in the high intensity treatment, 15 × 36 W 12% UV‐B fitted were fitted instead (G). The control plants and boxes were rotated between chambers fortnightly and plants in the low and high UV treatments were rotated and assorted within and across shelves within their assigned chambers. UV, ultraviolet.

For the long‐term UV exposure, 54 plants were grown in March 2018 from seed within two SANYO MLR‐351 H, Panasonic plant chambers (18 plants per UV intensity treatment; Figure 2). The total duration of the experiment was set at 75 days. The seeds were potted in pots (7 cm × 7 cm × 7 cm) containing compost and vermiculite (both Verve), and were randomly assigned to a UV intensity treatment (control, low, high, see Section 2.3).

For the short‐term exposure, *B. rapa* seeds were potted and grown (soil and pots used as above) in a glass greenhouse for 18 days prior to placing them in the chambers for 57 days (Figure 2), so the experiment was terminated when plants from both long‐term and short‐term exposure were 75 days old to allow meaningful comparisons. The short duration of UV exposure (~6 days) was defined as 25% of UV radiation (in days) received by the long exposure plants prior to flowering. Mean day of first flowering in the long‐term UV exposure experiment was day 24; therefore, we estimated that the plants should receive approximately 6 days of UV radiation in the growth chambers before flowering would begin. On day 18 of growth, 51 plants growing in the greenhouse were randomly assigned to UV treatment groups and transferred to the growth chambers (Figures 1 and 2). Daily Solarmeter® (Model 6.2 Sensitive UV‐B Meter) measurements indicated the greenhouse UV‐B irradiance was 0 W/m^2^ for the duration of growth prior to transfer to the plant chambers. The growth conditions were set as the above long‐term UV exposure experiment, and the plants were grown for a further 57 days.

### 2.3 Experimental design: UV‐B intensity treatments

Three UV radiation intensities were administered across two plant chambers (SANYO MLR‐351 H, Panasonic; Figure 2). Treatments were (i) control (no UV exposure), (ii) low UV exposure, and (iii) high UV exposure. Low UV intensity was achieved in one chamber using 15 × 36 W 6% UV‐B bulbs (ArcadiaReptile) and for the high UV intensity, a second chamber was fitted with 15 × 36 W 12% UV‐B bulbs (ArcadiaReptile). In both treatments, lamps provided in addition to 6% or 12% UV‐B the full light spectrum +30% UV‐A as specified by the supplier (ArcadiaReptile). These percentages represent the total spectral power output that falls within the ranges of UV‐B (280–315 nm) and UV‐A (315–400 nm) radiation (see supplier's website for more detailed information and graphical representations of the spectral power distribution of both bulbs). Both chambers had a control treatment; one custom‐built Perspex® VE grade UV‐absorbing acrylic box (*L* 45 cm × *W* 40 cm × *H* 40 cm) with an open top that permitted air flow was placed in the top shelf of both chambers (Figure 2). Control boxes blocked 99.99% of UV light transmission from 250 to 400 nm (as specified by the supplier) and contained 18 plants per box. UV‐B radiation was measured in the central point of each treatment shelf/box weekly with a Solarmeter® (Model 6.2 Sensitive UV‐B Meter). Mean daily UV‐B for the control treatments was 0.0027 ± 0.0027 W/m^2^, mean UV‐B for the low‐intensity chamber was 0.2072 ± 0.0198 W/m^2^, and mean UV‐B for the high‐intensity chamber was 0.3238 ± 0.0190 W/m^2^. For comparison, 0.74 W/m^2^ is about eight times greater than the level detected in midsummer in the UK (Baker et al., 1997). UV treatments were significantly different (*F*_2,30_ = 377.8, *p* < .001), and Tukey multiple pairwise comparisons indicated that all three UV treatments significantly differed from one another (all *p* < .001).

All plants were exposed to 12 h light/12 h dark cycle, 23°C light/20°C dark cycle, and 60% relative humidity within their chambers, and were watered every second day for the duration of the experiment. The control plants and boxes were rotated between chambers fortnightly and plants in the low and high UV treatments were rotated and assorted within and across shelves within their assigned chambers. The duration of flowering for all plants was recorded. It should be noted that all plants may have received some levels of UV‐A radiation before and during the experiment and the amount of PAR probably differed between plants grown exclusively in the chambers vs the greenhouse.

### 2.4 Flower collection and petal UV photography

In each long‐term and short‐term UV exposure plants, all flowers were removed upon anthesis, noting plant age (i.e., days since planting). The natural curvature of *B. rapa* petals made it difficult to accurately measure the UV‐absorbing part of the petals, so we removed the petals from each flower and pressed them in a plant‐press in the dark for at least 7 days, ensuring that all petals were adaxially presented and evenly flattened. Koski and Ashman (2013) indicated that this method of drying and pressing specimens to measure the size of UV‐absorbing area does not influence measurements, which we corroborated for our species too.

**FIGURE 3:**
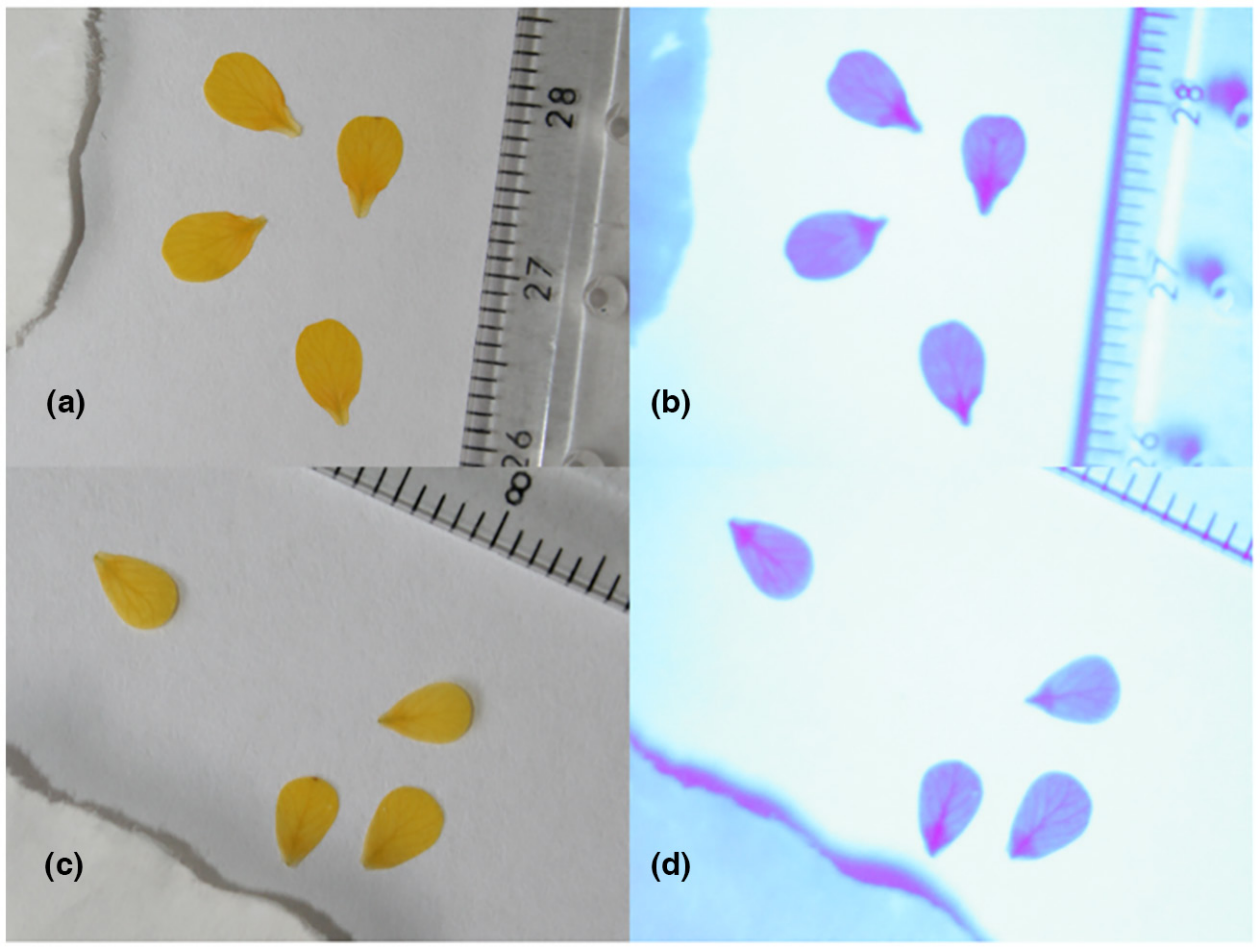
Petals from *Brassica rapa* experimental plants grown at low (a, b) and high (c, d) UV treatments photographed under visible light (a, c) and under UV‐A light (b, c). UV, ultraviolet.

A total of 726 pressed flowers were photographed in UV‐A light. All photographs were taken with a Canon EOS 400D camera, fitted with a Baader UBVRI 1¼′′ Photometric U‐filter. The filter permits the transmission of UV‐A light between 320 and 390 nm, peaking with 85% transmission at 350 nm, while removing infrared and daylight wavelengths. The availability of naturally occurring UV light in the daylight spectrum can be limited indoors; the image field was therefore illuminated with a UV‐A torch (Convoy S2+ Nichia UV waterproof LED flashlight) with a peak UV emission at 365 nm. The camera was mounted on a tripod and arm to achieve an overhead view of the pressed specimen. All photographs included a standard scale (i.e., ruler, Figure 3), and the UV torch was also positioned approximately 15 cm above the specimen. For all UV photographs, the exposure was 10 s, and the aperture f/5.6. Alongside each image, we include a piece of paper covered in sunscreen which appeared dark in images as it absorbed UV.

**FIGURE 4:**
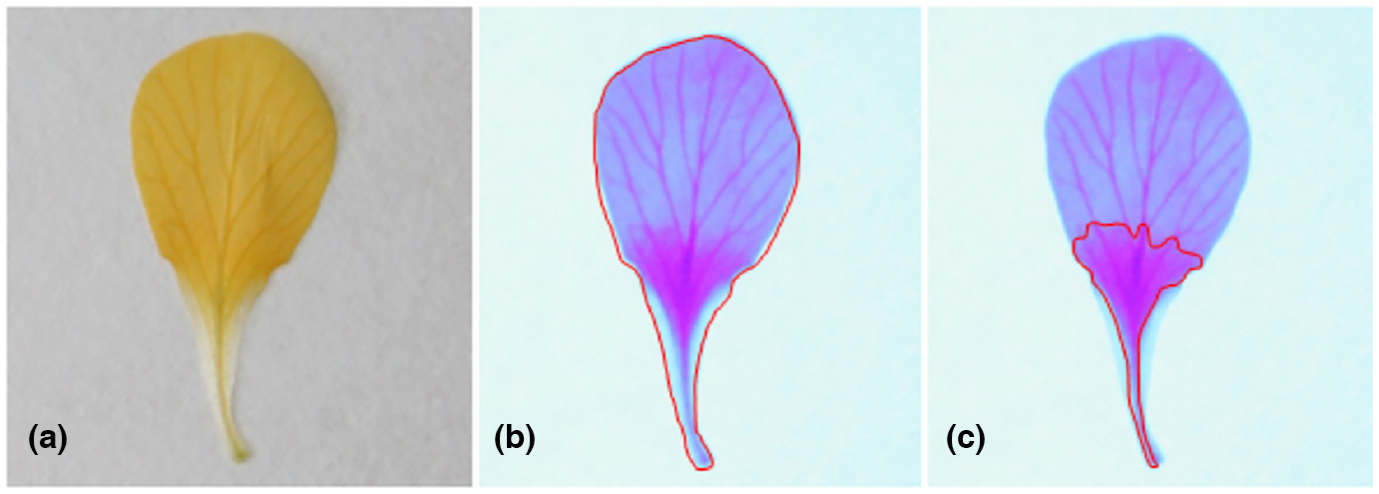
Pressed *Brassica rapa* petal in visible light (a) and UV light (b, c). The UV_proportion_ (%) of petals is the measure of (c) UV_area_ (mm^2^) relative to (b) total petal area (mm^2^). UV, ultraviolet.

From the 726 pressed flowers, the total UV‐absorbing area (UV_area_, mm^2^) and total petal area (mm^2^) could be accurately measured for a total number of 1871 petals from 644 different flowers from 70 plants, which could be photographed successfully after pressing the petals without damage and with clear focus of the petal (Table S1; Figure 3). All area measurements were made in Image J (2012, version Fiji, Schindelin et al., 2012) following Yoshioka et al. (2005) and Koski and Ashman (2013, 2016), with the petal UV_proportion_ calculated as the UV_area_/total petal area (Figure 4).

### 2.5 Statistical analysis

We first tested whether the total number of flowers and duration of flowering of each plant varied with UV‐intensity treatment (control, low, and high) and exposure length (short and long), using a two‐way Kruskal–Wallis test.

We then tested variation in UV_proportion_ and petal area, which were modeled using a linear mixed‐effects model (LMM), with individual plant included as a random effect, with flower as a random effect nested within individual plant. We first tested the relationship between radiation intensity treatment (control, low, and high), exposure length (short and long), and their interaction on UV_proportion_ and petal area. UV_proportion_ was logit‐transformed using the car package (Fox & Weisberg, 2019), to improve the normality of model residuals. Following this analysis, we also included plant age (days since planting) as a quadratic covariate to investigate further how this may affect petal responses to increase UV‐B. We included the quadratic relationship based on a reviewer's suggestion, and based on likelihood ratio tests, the models performed significantly better than plant age as a linear covariate. We again tested whether the inclusion of interactions between flowering date, treatment and exposure increased model fit, and the likelihood ratio tests supported the inclusion of the two‐way interactions over other model types. LMM modeling was carried out using the statistical package “lme4” (Bates et al., 2015) run in R version 3.6.3 (R Development Core Team, 2020). Post hoc testing between factors was carried out using the package “emmeans” (Lenth, 2018).

## 3 RESULTS

### 3.1 Effects of UV intensity and duration of exposure on flower production and duration

**FIGURE 5:**
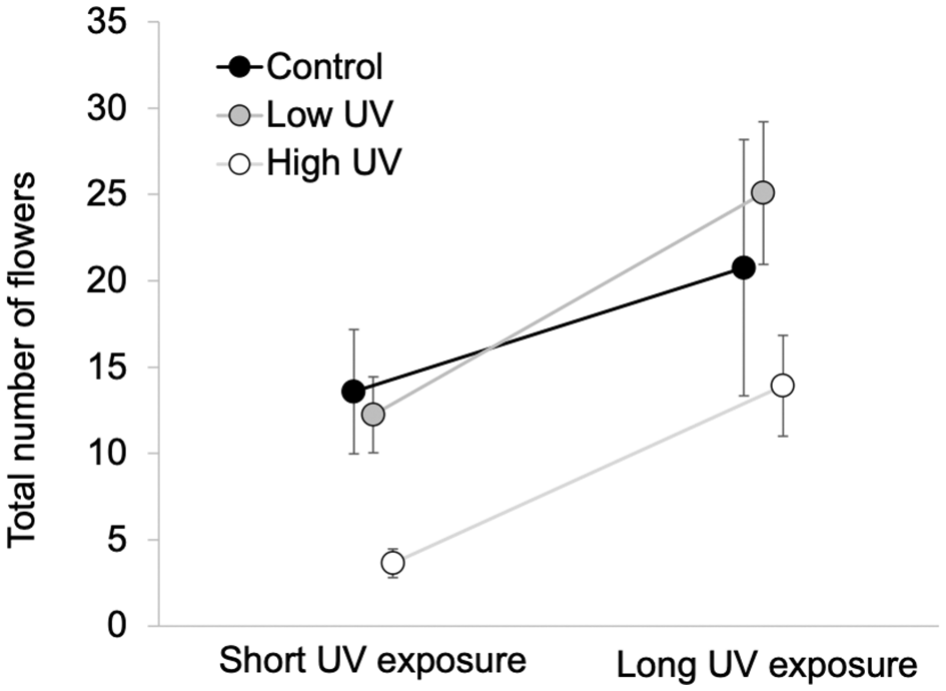
Mean ± SE number of flowers produced for control, low, and high UV intensity treatments *Brassica rapa* plants from short and long UV exposure treatments. SE, standard error; UV, ultraviolet.

There was a significant effect of both duration of exposure (*H* = 7.13, *p* < .001) and UV intensity treatment (*H* = 7.29, *p* = .026) on total flower numbers, without a significant interaction between these two factors (*H* = 3.64, *p* = .162; Figure 5). Long exposure plants produced on average twice as many flowers than plants grown with a short UV exposure, while plants grown in high UV intensity treatments produced significantly fewer flowers than both control and low UV treatments (Figure 5). In contrast, flowering duration was only statistically affected by UV intensity (*H* = 9.43, *p* = .009; *H* = 3.53, *p* = .060; and *H* = 1.22, *p* = .544, for the effect of intensity, duration, and the interaction between these two factors), with control and low UV intensity plants flowering longer than high UV intensity plants (32.8 ± 4.1, 28.8 ± 3.8, and 19.6 ± 3.1 mean ± standard error days for control, low, and high UV intensity respectively).

### 3.2 Changes in petal UV_proportion_ and petal area in relation to UV intensity and duration of exposure

**FIGURE 6:**
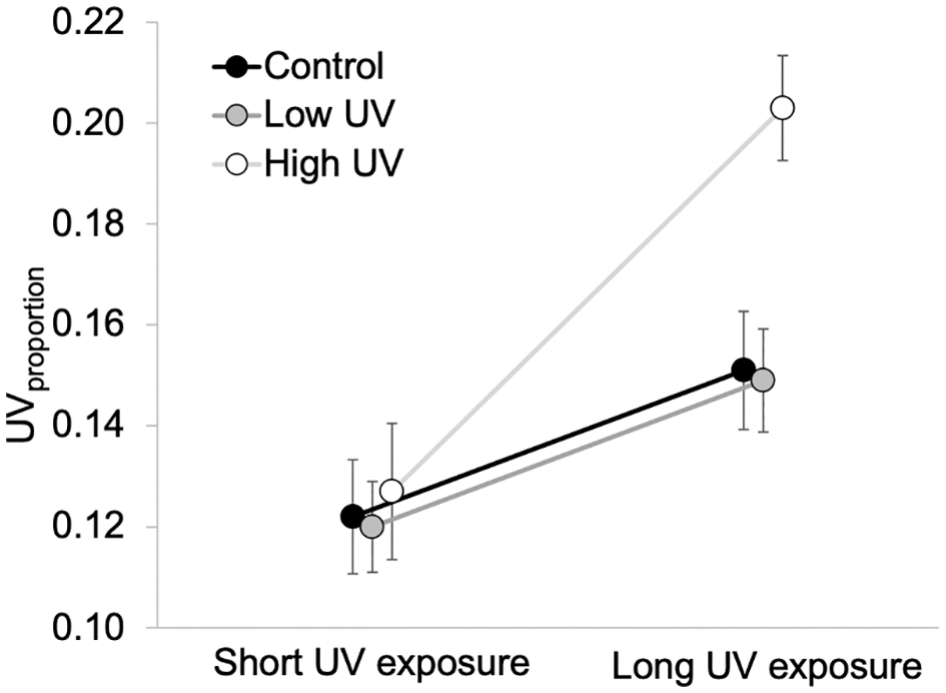
Estimated marginal means ± SE petal UV_proportion_ for control, low, and high UV intensity treatments and exposure (short, long) of *Brassica rapa* plants. SE, standard error; UV, ultraviolet.

There was a significant effect of UV intensity treatment and exposure on the UV_proportion_ of petals, but no significant effect of the interaction (Table 1). For long exposure plants, those grown in the high intensity treatments had significantly greater UV_proportion_ than both control (*t*‐ratio = −3.28, *p* = .005) and low intensity (*t*‐ratio = −3.68, *p* = .002) plants, with no significant difference between control and low intensity plants (*t*‐ratio = 0.13, *p* = .991; Figure 6). Among short exposure plants, there were no differences between intensity treatments detected (all *p* > .9). Overall, the UV_proportion_ between them was greater in long exposure plants compared to short exposure plants (Figure 6).

**TABLE 1:** Petal UV_proportion_ and petal area (mm^2^) in relation to UV exposure (short/long) and UV intensity treatment (control, low, and high).

Model	Parameter	*F* value	df	*p*
UV_proportion_	Intensity	3.80	2, 66.40	**.027**
	Exposure	21.94	1, 67.24	**<.001**
	Exposure × Intensity	−1.84	2, 66.40	0.167
Petal area	Intensity	0.79	2, 57.89	0.457
	Exposure	5.22	1, 57.89	**.026**
	Exposure × Intensity	0.75	2, 57.04	0.481

Significant values are indicated in bold.

Abbreviation: UV, ultraviolet.

For petal areas, there was a significant effect of exposure, but not UV intensity treatment (Table 1); the petals of short exposure plants were approximately 13% larger than in long exposure plants (10.55 ± 0.35 vs. 9.35 ± 0.38 mm^2^).

### 3.3 Changes in UV_proportion_ and petal area in relation to plant age

**FIGURE 7:**
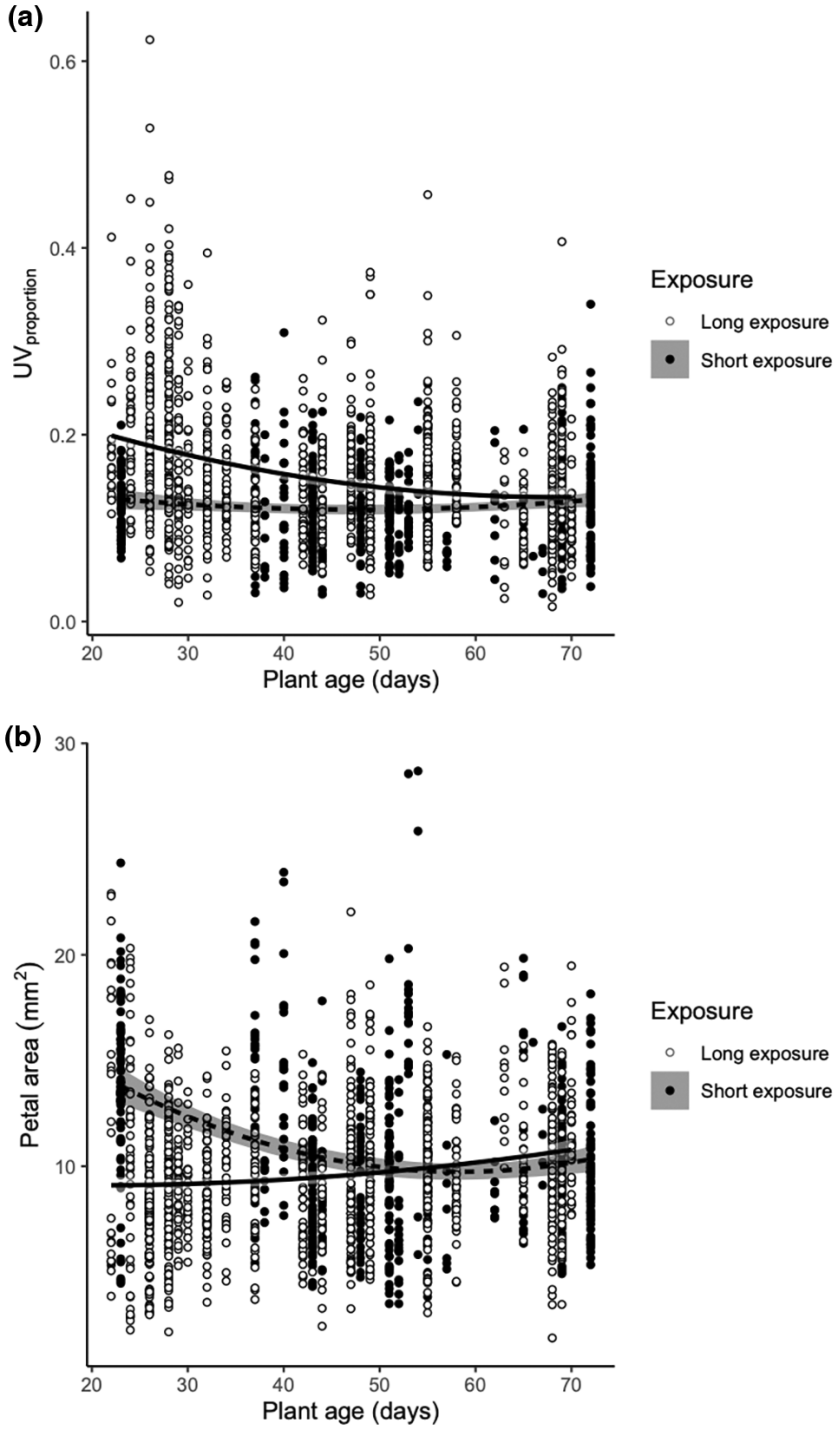
(a) UV_proportion_ in relation to plant age (days) for plants from short (filled circles, dashed line) and long UV exposure (white symbols, solid line) and (b) petal area (mm^2^) in relation to plant age (days) for plants from short (filled circles, dashed line) and long UV exposure (white symbols, solid line). UV, ultraviolet.

For UV_proportion_, there were significant two‐way interactions between plant age and exposure and plant age and UV intensity treatment (Table 2). Petals had significantly smaller UV_proportion_ as plants became older in long exposure plants, but not short exposure plants (Figure 7a). UV_proportion_ also became smaller with plant age in high intensity UV treatments, with a decrease also in low or control plants that was however not statistically significant (Figure S1).

**TABLE 2:** Petal UV_proportion_ and petal area (mm^2^) in relation to plant age and UV exposure (short/long) and intensity treatment (control, low, and high).

Model	Parameter	*F*	df	*p*
UV_proportion_	Exposure	18.20	1, 69.94	**<.001**
	Plant age (quadratic)	13.64	1, 612.47	**<.001**
	Treatment	3.86	2, 66.70	**.026**
	Plant age (quadratic) × Exposure	5.80	1, 617.51	**.003**
	Plant age (quadratic) × Treatment	2.99	4, 601.88	**.018**
Petal area	Exposure	6.64	1, 60.78	**.012**
	Plant age (quadratic)	8.40	2, 627.20	**<.001**
	Treatment	1.43	2, 57.78	.245
	Plant age (quadratic) × Exposure	20.82	2, 632.56	**<.001**
	Plant age (quadratic) × Treatment	6.42	4, 619.13	**<.001**

Significant values are indicated in bold.

Abbreviation: UV, ultraviolet.

For petal area, there were similar significant two‐way interactions between plant age and exposure, and plant age and UV intensity (Table 2). Petal area became smaller with plant age in short exposure plants, but not in long exposure plants (Figure 7b). Petal area also declined in high UV intensity treatments, but did not change in control or low UV intensity plants (Figure S2).

## 4 DISCUSSION

The reproductive structures of plants and especially pollen are sensitive to UV radiation (Feng et al., 2000). Several studies have shown that one of the most effective defensive mechanisms in flowers against UV‐induced stress is the accumulation of a wide range of UV‐absorbing phenolic compounds in the petals and in other structures such as pollen (Demchik & Day, 1996; Jenkins, 2009; Rozema et al., 1997). However, it is not clear whether plants can increase these UV‐absorbing plastically when in a high UV environment.

In this study, we found that *B. rapa* plants decreased flower production when grown with a short UV exposure and especially under high UV intensity, which also decrease the time they were flowering for. In addition, the petals of *B. rapa* plants grown in high UV intensity conditions had larger UV_proportion_ than low and control UV intensity plants. Areas of UV‐absorbing pigments at the base of petals (bullseyes) can potentially reduce reflected UV damaging pollen; larger bullseyes in artificial flowers provide enhanced protection to UV sensitive pollen, as compared with flowers with smaller UV bullseyes (Koski & Ashman, 2015). Thus, our findings suggest that the UV_proportion_ of *B. rapa* plants can respond plastically to increase UV absorption in response to UV radiation, demonstrating for the first time that petals are capable of plastic change in response to ambient UV radiation even after a relatively short exposure time.

We also found that longer exposure increased overall UV_proportion_ in petals. It is possible that increased duration of UV exposure early in plant growth (i.e., from seedling stage) may be important for the production of flavonoids that are responsible for the UV‐absorbing pigments in floral tissues. While UV exposure early in plant development may increase flavonoid accumulation in both vegetative tissues of *B. rapa*, and later in floral tissues, it is at present unclear by what mechanism exposure of vegetative tissues to UV affects later floral development (Musil & Wand, 1993, but see Llorens et al., 2015). Nevertheless, exposure to UV during seedling development may be important in altering floral tissue components (Yao et al., 2015).

Besides affecting UV_proportion_, UV intensity also affected petal size in our study. Overall petal area was smallest in long exposure treatments, while no significant difference was observed between UV intensity. In comparison, previous studies have reported a range of negative to positive effects or no changes in petal area in response to UV‐B (see Llorens et al., 2015 and references therein). As well as study‐specific differences (UV dose, exposure, and measurement), evidence suggests that how plants respond morphologically is also species specific and depends on the parameter measured. This is probably because UV‐mediated morphogenesis is the result of multiple compensatory molecular and physiological mechanisms that interact with other environmental variables (Barnes et al., 1990; Jansen et al., 2012, 2019).

Across low and high UV treatments, the UV_proportion_ was found to decline as plants aged in the long UV exposure treatment. This effect was not found in short UV exposure treatment. For many plants, there is a decrease in resource allocation and reproductive investment over the flowering period that leads to smaller floral structures, and fewer gametes, and seeds (Ashman & Hitchens, 2000; Kliber & Eckert, 2004) and in long exposure plants, this decline in UV_proportion_ suggests some sort of costs of general reproduction. The fact there is no change in short exposure plants suggests that UV intensity may be an additive cost.

When looking at the effect of plant age on petal area, significant effects were only detected across exposure length and intensity treatment. Controls appeared to show no change over time, whereas long exposure plants seem to show an increase in petal area over time, and in the short exposure group there was no change over time or slight decrease. It is not clear why this was the case, as declines in flower size are normal with plant age in related species (e.g., Williams & Conner, 2001). It may be two differing time‐dependent responses; plants can respond rapidly to UV stress (i.e., hours, Greenberg et al., 1996), the observed increase is likely a time lagged response between stress and effect on phenotype, as UV‐exposed plants balance the damage, repair, acclimation, and adaptation responses at different molecular and physiological levels, which require different tempos (Jansen et al., 1998), translating into responses to UV modulated by organ/organism ontogeny.

## 5 CONCLUSIONS

The plastic responses of floral UV‐absorbing area demonstrated in this study indicate the potential for plants to persist when faced with environmental UV‐B change by decreasing flower production but enhancing petal UV_proportion_ in these flowers. We identified a significant positive correlation between the intensity and duration of UV exposure and UV_proportion_ in *B. rapa*. Even though not tested specifically here, these responses may be important for the hypothesized reduction in exposure to UV radiation of the floral reproductive structures caused by a reduction in the proportion of reflected UV radiation that they receive as a consequence of the increased UV bullseye. Moreover, our study demonstrates that flowers can potentially acclimate to different UV radiation intensities and duration of exposure through an increase in UV‐absorbing areas. Such a rapid plastic response may be especially beneficial in regard to climate change, even though field studies involving multiple species and environmental factors should be employed to fully understand the complexity of UV response in plants.

## CONFLICT OF INTEREST

The authors declare no conflict of interests.

## Supporting information


Appendix S1

## Data Availability

The data that support the findings of this study are available at FigShare (https://doi.org/10.6084/m9.figshare.21202628.v2; Gray et al., 2022).
