# Supplementary material for: Floral ultraviolet absorbance area responds plastically to ultraviolet irradiance in Brassica rapa
Source: Plant Environ Interact. 2022 Sep 29;3(5):203–11. doi: 10.1002/pei3.10091 (PMC10168085; doi:10.1002/pei3.10091)
Supplement: Supplementary file 1 — Appendix S1 [file PEI3-3-203-s001.docx]

**Table S1**. Number of plants, flowers, and petals measured in the study for long- and short-term UV exposed *Brassica rapa* grown in control, low, or high UV intensity.

|  |  | **Control** | **Low** | **High** |
| --- | --- | --- | --- | --- |
| **Long term** | Plants | 10 | 12 | 12 |
|  | Flowers | 130 | 168 | 116 |
|  | Petals | 380 | 508 | 359 |
| **Short term** | Plants | 11 | 14 | 11 |
|  | Flowers | 95 | 105 | 30 |
|  | Petals | 249 | 296 | 79 |

**Figure S1.** The relationship between UV_proportion_ and plant age (days), for control (open circle, solid line), low UV intensity (black circles, long dashed line) and high UV intensity (grey circles, short dashed line).

**Figure S2.** The relationship between petal area (mm^2^) and plant age (days), for control (open circle, solid line), low UV intensity (black circles, long dashed line) and high UV intensity (grey circles, short dashed line).
